# Gender-Related Clinical Characteristics in Children and Adolescents with ADHD

**DOI:** 10.3390/jcm11020385

**Published:** 2022-01-13

**Authors:** Pietro De Rossi, Italo Pretelli, Deny Menghini, Barbara D’Aiello, Silvia Di Vara, Stefano Vicari

**Affiliations:** 1 Child and Adolescent Neuropsychiatry Unit, Department of Neuroscience, Bambino Gesù Children’s Hospital, IRCCS, 00146 Rome, Italy; pietro.derossi@opbg.net (P.D.R.); italo.pretelli@opbg.net (I.P.); deny.menghini@opbg.net (D.M.); barbara.daiello@opbg.net (B.D.); silvia.divara@opbg.net (S.D.V.); 2 Department of Human Science, LUMSA University, 00193 Rome, Italy; 3 Department of Life Science and Public Health, Università Cattolica del Sacro Cuore, 00168 Rome, Italy

**Keywords:** ADHD, gender, characteristics, boys, girls, CBCL, CPRS

## Abstract

Attention Deficit/Hyperactivity Disorder (ADHD) is the most frequently diagnosed neurodevelopmental disorder in school-age children, and it is usually associated with a significant impairment in global functioning. Traditionally, boys with ADHD are more likely to be referred for clinical assessments due to a higher prevalence of externalizing symptoms. However, as regards gender-related differential clinical characteristics between boys and girls with ADHD, further investigation is warranted in light of conflicting results found in currently available literature. In fact, a more precise clinical characterization could help increase appropriate diagnoses and treatment planning. In this context, we carried out a retrospective observational study on 715 children and adolescents diagnosed with ADHD from 2018 to 2020 at our center, in order to describe their gender-related clinical characteristics. Boys displayed higher average IQs, but they were comparable to girls in functional impairments and adaptive skills. Girls displayed higher scores on the Attention Problems subscale of the CBCL 6–18 and on several CPRS-R:L subscales, suggesting higher general ADHD symptom severity. Boys showed higher scores on CBCL 6–18 subscales, such as withdrawn/depressed, internalizing, and obsessive-compulsive problems. In conclusion, girls showed more severe ADHD features and lower IQ in clinically referred settings, while boys showed more internalizing problems and obsessive-compulsive symptoms.

## 1. Introduction

Attention deficit and hyperactivity disorder (ADHD) is one of the most common neurodevelopmental disorders diagnosed in early childhood and adolescence. According to DSM-5, it is characterized by altered and unusual levels of inattention and hyperactivity compared to what is observed in typical child development [1]. ADHD symptoms usually determine functional impairment in familiar, academic, and social context.

ADHD worldwide prevalence in school-age children is 5.3% [2]. In Italy, a prevalence range between 1.1% and 3.1% is estimated among children and adolescents aged 5 and 17 years, with boys displaying a prevalence rate 1.2–7.6 higher than girls [3]. It may be noticed that this is lower than the estimated worldwide prevalence, and this is probably due to methodological and cultural factors that are addressed within the Italian prevalence study [3].

A representative Danish survey based on health registry data collected from 1995 to 2010 reported that ADHD incidence rates increased by a factor of approximately 12 during this period. Furthermore, it was also reported that the male-to-female ratio decreased from 7.5:1 to 3:1 among school-age children and from 8.1:1 to 1.6:1 among adolescents [4]. These data probably reflect an increased awareness of ADHD symptoms, globally, and specifically in girls. In other countries, it is assumed that girls are still underdiagnosed [5].

Traditionally, boys are more likely to be referred, diagnosed, and treated for ADHD symptoms than girls. This seemed to depend on gender differences in symptomatology: for example, males would have more disruptive/externalizing symptoms [6,7], which would alert diagnostic evaluations earlier than females.

This was confirmed by a recent publication, as boys showed higher impulsivity compared to girls while girls displayed higher levels of inattention compared to boys. However, the same study found no differences in the hyperactivity and distractibility levels of boys and girls [8], suggesting that gender differences in ADHD phenotypical presentations should be better studied in order to overcome the diagnostic/therapeutic gap described above.

As regards a gender-specific comorbidity pattern in ADHD, the available literature generally supports a higher prevalence rate of externalizing disorders (conduct disorder, oppositional defiant disorder) and symptoms (e.g., aggression, rule-breaking) in boys, and a higher prevalence rate of internalizing disorders (e.g., anxiety) in girls [9,10].

In this context, our aim is to describe gender-related clinical characteristics of 715 children and adolescents diagnosed with ADHD from 2018 to 2020 in a retrospective observational study. Our results will be discussed in light of the existing international literature, in order to provide additional pieces of evidence, helping to reduce the significant gender-related differences in timely and effectively diagnosing and treating of ADHD in developmental age.

## 2. Materials and Methods

### 2.1. Participants

In this retrospective study, 715 drug-naïve children and adolescents with ADHD who attended the Child and Adolescents Neuropsychiatry Unit of the Bambino Gesù Children’s Hospital (Rome, Italy) for a first diagnosis were recruited over the course of 3 years (from January 2018 to December 2020).

Children and adolescents (mean age (years) = 9.4, SD = 2.9; 108 girls with ADHD and 607 boys with ADHD) received their first diagnosis of ADHD by experienced developmental psychiatrists and neuropsychologists, according to the fifth edition of the Diagnostic and Statistical Manual of Mental Disorders (DSM-5) criteria [1].

Only patients without comorbidities were selected from all the cases assessed at our center and finally entered in the final sample of 715 children and adolescents previously described in detail. This choice was made in order to increase homogeneity in results, as the comorbid group was too small and heterogeneous due to the presence of multiple/complex comorbid conditions. Furthermore, patients were included in the study if they met the following criteria: (a) the absence of neurological and neurosensory deficit; (b) the absence of autism spectrum disorder; (c) the absence of past drug treatment; (d) the absence of intellectual disability.

Psychiatric diagnoses were based on developmental history, extensive clinical examination, and the Schedule for Affective Disorders and Schizophrenia for School-Age Children—Present and Lifetime Version, DSM-5 [11], a semi-structured interview that assesses the presence of psychopathological disorders according to a DSM-5 classification.

All participants and parents were informed about assessment instruments and treatment options. Written informed consent was obtained from parents. The study was conformed to the Declaration of Helsinki.

### 2.2. Instruments

Global functioning was assessed with the Children’s Global Assessment Scale (C-GAS) [12]. The C-GAS estimates the overall severity of disturbance (range = 0–100). Scores over 90 indicate superior functioning, whereas scores under 70 suggest impaired global functioning.

IQ was measured by using the Wechsler Intelligence Scale for Children-IV (WISC-IV) [13] or Colored Progressive Matrices or Standard Progressive Matrices (CPM, SPM) [14]. The global IQ was considered in the analysis (M = 100, SD = 15).

The adaptive skills were evaluated by means of The Adaptive Behavior Assessment System—Second Edition (ABAS-II) [15] or the Vineland Adaptive Behavior Scales—Second Edition (Vineland II) [16]. ABAS-II provides a comprehensive norm-referenced assessment of the adaptive skills. The norm-referenced standard scores of ABAS-II of the General Adaptive Composite (GAC) was considered in the analysis (M = 100, SD = 15). The Vineland II also measures personal and social skills needed in an individual’s everyday life. The Adaptive Behavior Composite was considered in the analysis (M = 100, SD = 15).

Conners’ Parent Rating Scales Long Version Revised (CPRS-R:L) [17] was used to assess behaviors related to ADHD. It is completed by parents to obtain a measure of hyperactivity and inattention symptoms for ADHD; it comprises 14 subscales. It generates a T-score for each subscale. The cutoff for T-scores for clinical significance is >70 (very elevated) and T-scores from 60–70 are considered as high averages or elevated.

The Child Behavior Checklist for Ages 6–18 years (CBCL 6–18) [18] is completed by parents and is a questionnaire of child and adolescent behaviors and emotions. It generates a T-score for each subscale. According to normative data, a T-score above 64 is considered significant for the three broadband scales, whereas for the syndrome scales, the cut-off for clinical significance is 70. 

### 2.3. Statistical Analysis

When data were normally distributed and the assumption of homogeneity was not violated, parametric tests were computed. The Shapiro–Wilk test was used to test the normality of the data and Levene’s test for the homogeneity of variances. Student’s t-tests were used to compare the boys vs. girls groups on age, IQ, ABAS-II, and C-GAS. 

Multivariate analysis of variance (MANOVA) was used to compare the boys and girls groups on CBCL scales and on CPRS-R:L scales. Considering that the two groups differed in IQ, MANCOVA on CBCL scales and on CPRS-R:L scales were conducted, also controlling for IQ. The Fisher LSD test was used for post-hoc analyses on CPRS-R:L subscales, CBCL 6–18 subscales, Group x CPRS-R:L subscales, and on Group x CBCL 6–18 subscale effects. Box’s M test was used to check the equality of multiple variance–covariance matrices.

The statistical software SPSS Version 22 (IBM Corporation, 2017) was used for analyses. 

## 3. Results

Boys with ADHD did not differ from girls with ADHD for age in years (t_711_ = −0.75, *p* = 0.45; boys: M = 9.48, SD = 2.89, range = 6–18; girls: M = 9.87, SD = 11.12, range = 6–16), global functioning (C-GAS) (t_458_ = −0.72, *p* = 0.46; boys: M = 52.93, SD = 7.15; girls: M = 53.63, SD = 6.57) and adaptive skills (t_670_ = 0.43, *p* = 0.66; boys: M = 71.74, SD = 16.14; girls: M = 70.98, SD = 15.58). However, boys with ADHD displayed higher IQ compared to girls (t_547_ = 2.08, *p* = 0.03; boys: M = 105.34, SD = 18.00; girls: M = 100.71, SD = 20.36).

ADHD symptoms (CPRS-R:L) of boys and girls were compared by means of a MANCOVA with 14 CPRS-R:L subscales, within factor and group (boys vs. girls) as between, controlling for IQ (to take into account the difference in IQ between boys and girls with ADHD). The group effect was not significant (F_1, 525_ = 52.16, *p* = 0.06; boys: M = 67.02, SD = 10.23; girls: M = 69.98, SD = 10.43), while CPRS-R:L subscale effect (F_13, 6825_ = 25.51, *p* < 0.0001; η^2^_p_ = 0.04), and the Group x CPRS-R:L subscale interaction effect (F_13, 6825_ = 6.87, *p* < 0.00001; η^2^_p_ = 0.01) were both significant. Post-hoc analysis (Fisher LSD test) on CPRS-R:L subscales effect demonstrated that all participants showed higher scores in the ADHD index (mean = 78.23, SD = 0.82) than all other CPRS-R.L subscales (*p* < 0.001), with the exception of the DSM IV hyperactive/impulsive subscale (mean = 76.69, SD = 0.88). All participants showed higher scores in the DSM IV hyperactive/impulsive subscale (mean = 76.69, SD = 0.88) than all other CPRS-R.L subscales (*p* < 0.001), with the exception of the DSM-IV inattentive subscale (mean = 76.62, SD = 0.86).

Post-hoc analysis (Fisher LSD test) on Group x CPRS-R:L subscale effects showed that girls displayed higher scores than boys in several CPRS-R:L subscales, see Table 1. Box’s M test was non-significant (*p* > 0.001).

To evaluate differences between boys and girls in behavioral and emotional symptoms (CBCL 6–18), a MANCOVA was conducted with 20 CBCL 6–18 subscales, within factor and group (boys vs. girls), as between factor, controlling for IQ (to take into account the difference in IQ between boys and girls with ADHD). No group effect was found (F_1, 482_ = 29.62, *p* = 0.34). However a significant CBCL 6–18 subscale effect, (F_19, 9158_ = 14.49, *p* < 0.0001; η^2^_p_ = 0.02), and interaction Group x CBCL 6–18 subscale effects (F_19, 9158_ = 2.60, *p* = 0.0001; η^2^_p_ = 0.005) were found. Post-hoc analysis (Fisher LSD test) on the CBCL 6–18 subscale effect documented higher scores in attention problems (mean = 68.47, SD = 9.44) than in ADHD problems (mean = 67.69, SD = 8.23), and total problems (mean = 66.41, SD = 8.59; *p* always < 0.001). Post-hoc analysis also showed higher scores in affective problems (mean = 64.98, SD 9.00) than social problems (mean = 64.18, SD = 8.52), anxiety problems (mean = 64.05, SD = 7.89), thought problems (mean = 63.46, SD = 9.32), oppositional–defiant problems (mean = 63.29, SD = 8.44), anxious/depressed (mean = 63.19, SD = 9.01), conduct problems (mean = 62.91, SD = 8.87), internalizing problems (mean = 62.48, SD = 9.65), rule-breaking behavior (mean = 62.26, SD = 8.33), withdrawn/depressed (mean = 62.14, SD = 9.51), obsessive-compulsive problems (mean = 61.70, SD = 9.87), sluggish cognitive tempo (mean = 60.71, SD = 8.24), somatic complains (mean = 58.91, SD = 7.81), and somatic problems (mean = 57.32, SD = 7.93; *p* always <0.05). 

Post-hoc analysis (Fisher LSD test) on Group x CBCL 6–18 subscale effects showed that boys displayed higher scores in several subscales, see Table 2. Box’s M test was non-significant (*p* > 0.001).

## 4. Discussion

This retrospective observational study investigated gender-related clinical characteristics on a group of 715 children and adolescents at their first diagnostic assessment for ADHD. On the epidemiological level, our results are in line with international literature [19,20,21], with a male to female ratio of approximately 6:1. 

Our results showed that boys and girls with ADHD differed for IQ, but they were comparable for functional impairments or adaptive skills. Taking into account the difference in IQ, comparisons between girls and boys on behavioral and psychopathological characteristics showed that girls with ADHD obtained higher scores on the Attention Problems subscale of the CBCL 6–18 and on several CPRS-R:L subscales, suggesting higher general ADHD symptom severity. However, boys showed higher scores on CBCL 6–18 subscales, such as withdrawn/depressed, internalizing, obsessive-compulsive problems, related to mood and internalizing problems.

Previous findings have suggested that clinically diagnosed males and females usually showed similar symptom severity, except for higher inattention in females [22,23,24]. Our findings partially confirmed this evidence, as females in our sample displayed, besides inattention, higher general ADHD symptom severity. 

This could be at least partially explained by a referral bias, as it is possible that only the most severe girls were referred for early assessment and diagnosis at our ward, because predominantly inattentive aspects were generally harder to detect and less disturbing in the classroom or at home [25]. Moreover, our findings differed from previous studies, which found more mood and internalizing symptoms in girls than in boys [26,27]. 

However, recent findings by Slobodin and Davidovitch [8] are in line with our study, as they found more psychiatric internalizing co-occurring symptoms in boys as compared to girls. Alternatively, it is possible that the externalizing symptoms of boys were associated with elevated levels of emotional dysregulation [28,29] and, therefore, described as anxiety and depression.

As already mentioned, in our sample, boys with ADHD displayed a slightly higher IQ than girls. In turn, girls showed more pronounced ADHD features. However, this did not reflect higher functional impairments or deficits in adaptive skills in girls as compared to boys. With respect to this, on the one hand, the difference in IQ could be accounted for by the fact that girls are more frequently assessed, diagnosed, and treated either when they present with extremely severe ADHD global symptoms or when they suffer from prominently impaired inattention [30]. On the other hand, it is possible that girls with ADHD are (or become) better than boys at camouflaging or compensating their struggles [31], so that their functioning levels, as reported by parents or assessed by clinicians, may appear in our sample as comparable to boys, despite the significant difference in IQ and symptom levels.

Therefore, a picture emerged where the most significant differences between boys and girls with ADHD, within the clinically referred population, were quintessentially on behavioral and emotional symptoms. Among these differential symptomatological features, we found significantly higher levels of obsessive-compulsive symptoms in boys than in girls. The obsessive-compulsive dimension is usually characterized by intrusive thoughts and the need for compulsion. Further, it is usually associated with an inhibited temperament that is generally characterized by behavioral restraint, withdrawal, and avoidance of novel stimuli [32,33]. At the same time, compulsions could be associated with an impulsive and risky behavioral profile [34]. 

Finally, ADHD and obsessive-compulsive dimensions share some common neurobiological features, including dysfunctions in cortical–striatal–pallidal–thalamic circuits [35], and are more frequently present in boys during childhood [36]. In this light, it seems reasonable that our boys with ADHD, characterized by higher levels of internalizing symptoms, also displayed higher levels of obsessive-compulsive symptoms. Furthermore, obsessive-compulsive symptoms in boys with ADHD have to be carefully assessed as they could transiently increase during initial phases of up-titration, in case of methylphenidate treatment [37].

Traditionally, evidence emerging from studies on gender-related clinical characteristics in the normal population is usually consistent with a higher prevalence of internalizing symptoms, such as behavioral inhibition, worry, and anxiety symptoms, in girls [38,39]. This is in line with data on clinically diagnosable psychiatric disorders within the general population [40]. Furthermore, a recent study showed a specific association between internalizing behaviors and microstructural brain characteristics in typically developing girls [41]. Conversely, in our study, internalizing symptoms are more pronounced in boys, raising the possibility that ADHD may exacerbate or anticipate internalizing problems, such as anxiety and depression, by conferring boys a vulnerability based on difficulties in global and relational functioning.

The main limitation of our study is that we did not assess characteristics in the general population that were not clinically referred, where ADHD seems to be more represented in females than it usually is in clinical samples [30]. For this reason, our study may suffer from a selection bias towards a group of girls with severe ADHD, and this could have had a significant impact on our comparison of clinical differential characteristics of girls and boys with ADHD.

Another potential limitation is that consistent and reliable information on ADHD diagnosis of the parents or other clinically significant data regarding parents were not available in our dataset. This is also because ADHD is rarely diagnosed in adults in Italy, and it was rarely diagnosed in developmental age when the parents of our patients were children. Therefore, it is possible that at least part of the parents have undiagnosed persistent adult ADHD.

We acknowledge that our paper reports on a much-studied topic in ADHD; for this reason, its originality for readers may be considered limited. Nonetheless, we reckon that it provides a very large sample of drug-naïve children and adolescents with ADHD at their first evaluation/diagnosis. Furthermore, our results highlight the fact that internalizing symptoms in a clinically referred sample may be more pronounced in boys, which is, in our opinion, something that should not be overlooked by clinicians in order to properly tailor multimodal treatment strategies to patients.

As for the potential differential impact of pharmacological and non-pharmacological treatments on gender-based clinical characteristics, follow-up studies on the same sample are ongoing. These studies will also help understand the potential gender-related impacts of treatment after 3 and 6 months of methylphenidate in the most severe cases needing pharmacological treatment. 

Finally, a distinct paper on age dependence of psychopathological characteristics of children and adolescents with ADHD is in preparation. In that context, the hypothesis that the aforementioned characteristics could differ by age within each gender group will be tested.

## 5. Conclusions

In conclusion, our study provided evidence that girls showed more severe ADHD features and lower IQ in a clinically referred setting, while boys showed more prominent internalizing problems and obsessive-compulsive symptoms.

Our results are important for clinicians, to consider evaluating and treating young children with ADHD. Indeed, girls with ADHD who are clinically referred for evaluation may display significantly severe presentations, particularly inattention, in which pharmacological treatment with methylphenidate is often warranted. On the other hand, internalizing symptoms should not be overlooked in boys with ADHD, as they could be specifically targeted by cognitive–behavioral treatment along with ADHD symptoms. 

Further studies with longitudinal designs are needed in order to establish whether (and how) pharmacological and non-pharmacological treatments for ADHD could positively impact gender-based differential clinical characteristics in the modeling of the developmental trajectory of the disorder.

## Figures and Tables

**Table 1:** Results of comparison between boys and girls with ADHD in CPRS-R:L scores.

CPRS-R:L Subscale	Boys	Girls	Fisher LSD Test
	Mean (SD)	Mean (SD)	*p*-Value
Oppositional	67.26 (16.15)	67.70 (15.20)	0.81
Cognitive problems/inattention	72.04 (14.33)	78.84 (15.31)	0.0002
Hyperactivity	70.34 (15.38)	75.61 (18.14)	0.004
Anxious/shy	57.45 (14.40)	57.84 (13.88)	0.83
Perfectionism	55.71 (14.00)	55.61 (12.42)	0.95
Social problems	65.39 (18.64)	62.41 (17.12)	0.1
Psychosomatic problems	56.14 (15.77)	54.42 (13.66)	0.35
ADHD Index	74.45 (13.22)	82.00 (14.75)	0.00004
Global index restless/impulsive	70.45 (13.85)	75.57 (14.43)	0.005
Global index emotional liability	62.08 (16.37)	61.94 (14.45)	0.94
Global index total	70.09 (14.85)	73.94 (14.02)	0.045
DSM-IV inattentive	73.21 (14.54)	80.02 (15.03)	0.0002
DSM IV hyperactive/impulsive	69.72 (14.28)	74.45 (16.36)	0.01
DSM IV total	73.84 (14.13)	79.54 (15.74)	0.002

**Table 2:** Results of comparison between boys and girls with ADHD on CBCL 6–18 scores.

CBCL 6–18 Subscale	Boys Mean (SD)	Girls Mean (SD)	Fisher LSD Test *p*-Value
Anxious/depressed	63.49 (9.04)	61.39 (8.62)	0.06
Withdrawn/depressed	62.57 (9.54)	59.66 (8.96)	0.01
Somatic complaints	58.84 (7.82)	59.29 (7.75)	0.69
Social problems	64.27 (8.43)	63.57 (9.03)	0.53
Thought problems	63.56 (9.28)	62.87 (9.56)	0.54
Attention problems	68.07 (9.48)	70.76 (8.90)	0.01
Rule-breaking behavior	62.36 (8.52)	61.61 (7.08)	0.50
Aggressive behavior	65.99 (10.71)	65.02 (8.68)	0.39
Internalizing	62.82 (9.38)	60.46 (10.90)	0.03
Externalizing	64.29 (9.50)	64.04 (8.19)	0.82
Total problems	66.51 (8.67)	65.83 (8.16)	0.54
Affective problems	65.15 (9.10)	63.92 (8.36)	0.28
Anxiety problems	64.14 (7.96)	63.47 (7.51)	0.55
Somatic problems	57.26 (7.91)	57.61 (8.07)	0.75
ADHD Problems	67.38 (8.37)	69.47 (7.15)	0.06
Oppositional defiant problems	63.42 (8.58)	62.49 (7.57)	0.41
Conduct problems	62.76 (9.09)	63.78 (7.43)	0.36
Sluggish cognitive tempo	60.82 (8.30)	60.00 (7.80)	0.46
Obsessive-compulsive problems	61.44 (9.87)	59.08 (9.65)	0.03
Post-traumatic stress problems	66.35 (8.91)	65.25 (8.87)	0.33

## Data Availability

The data presented in this study are available upon request from the corresponding author. The data are not publicly available due to privacy and ethical restrictions.
